# Waviness of Freeform Surface Characterizations from Austenitic Stainless Steel (316L) Manufactured by 3D Printing-Selective Laser Melting (SLM) Technology

**DOI:** 10.3390/ma13194372

**Published:** 2020-09-30

**Authors:** Tomasz Kozior, Jerzy Bochnia, Paweł Zmarzły, Damian Gogolewski, Thomas G. Mathia

**Affiliations:** 1 Department of Mechanical Engineering and Metrology, Kielce University of Technology, al. Tysiąclecia Państwa Polskiego 7, 25-314 Kielce, Poland; jbochnia@tu.kielce.pl (J.B.); pzmarzly@tu.kielce.pl (P.Z.); dgogolewski@tu.kielce.pl (D.G.); 2 Laboratoire de Tribologie et Dynamique des Systemes (LTDS)—CNRS Ecole Centrale de Lyon, 69134 Lyon, France; Thomas.Mathia@ec-lyon.fr

**Keywords:** freeform surface, 3D printing, waviness, rapid prototyping, surface topography, SLM technology

## Abstract

The paper presents the results of tests of surface waviness of samples made in the powder bed fusion technology. The models were built using 316L steel-based powder with high corrosion resistance. The samples were placed on the construction platform at three different angles (0°, 45°, 90°) in XZ plane. Then, using an optical profilometer, the parameters of the geometric structure of the surface of the primary profile and the separated waviness component were measured. Analyzing the results of the test, it can be stated that the orientation of model arrangement has an impact on the quality of the technological surface texture, what has significance impact on wear processes and mechanical properties.

## 1. Introduction

Additive manufacturing technologies have revolutionized the current rapid prototyping market. Thanks to a wide range of materials, both plastics, ceramics and metals, they can be used to build models in almost all branches of the industry. These technologies are widely used in the metal works industry (for the construction of casting models and molds) [1,2], medical industry (production of injection molds-SLM and SLS). Moreover, the layered technologies utilizing metal-based materials are widely used in the production of prototypes and fully functional components in the automotive and aviation industries, where the key factor is the low mass of manufactured components [3]. Moreover, it is possible to produce free-form surfaces using additive technologies. Currently, metal powder-based technologies are used in the dental industry, where the production of small, durable and corrosion-resistant prostheses is required [4,5]. The use of additive technologies is becoming more and more popular in the medical industry. To a large extent it is related to the possibility of using three-dimensional models obtained directly from X-rays and computed tomography. At the same time, there are scientific papers being published in which the creation of hybrid solutions is discussed, i.e., printing model based on existing objects, which is widely used in the textile industry [6], heavy industry [7] and filtering application [8,9]. Additive manufacturing itself has become a fully valuable field of science, in which new materials, technological parameters, dimensional and shape accuracy of manufactured objects are still being studied, in particular including the technological surface layer.

Despite the short time that has elapsed since the invention of the first layered technology (stereolithography) [10], in the following years there was an unimaginably vast development of physical objects based on layered structure. In less than 20 years, new and different technologies have been invented, differing both in the form of the input material and the way the layers are joined together. In particular, it is worth to mention one of the best known commercially used Fused Deposition Modeling (FDM) technology [11], Fused Filament Fabrication (FFF) [12], Selective Laser Sintering-SLS [13], Laminated Object Manufacturing (LOM) [14], Selective Laser Melting (SLM) [15] or 3D printing (3DP) [16]. In addition, ecological technologies such as FEF [17] have emerged in the last few years.

Analysing the current state of the art, it should be noted that there are papers evaluating the possibility of using additive technologies in terms of dimensional and shape accuracy, as well as quality of the surface texture [18,19]. Research on the influence of technological parameters on the quality of the geometric structure of the surface have been described in several research papers. The authors, however, largely focus their research on determining the parameters of the geometric structure of the surface (both 2D and 3D), without analyzing the waviness of the surface. This is all the more important since waviness plays a key role in the case of cooperating machine elements and it has an impact on their wear, which is an important parameter of the surfaces of the manufactured elements.

Surface waviness is periodically repeated irregularities where the ratio of the distance between the vertices of the irregularities to their height is equal to at least 40.

Excessive surface waviness is largely the result of improperly performed the manufacturing process. The surface waviness is formed as a result of vibration and flexibility of machine tools, whose inflexibility cannot be controlled. In [20], a model of surface waviness formation in parallel grinding process is presented. It was assessed that grinding wheel vibration significantly affects the generated surface waviness. Similar studies were presented by Wu in article [21]. The impact of polishing parameters on surface waviness of blisk blade in jet engines was assessed here. The influence of surface waviness is especially visible in the bearings. In papers [22] it has been shown that the waviness of 6304 type ball bearing raceways significantly affects the values of generated vibrations. In the article [23] the negative impact of the surface waviness of aerostatic bearings on the reduction of dynamic stiffness and dynamic damping were assessed. Measurement of surface waviness is important in the paper and cellulose industry [24]. It was found in [25] that the waviness value of steel surfaces affects the quality of paint location.

It should be added that the surface waviness is a more wide-scale phenomenon, hence its heterogeneity in many cases. Analysing the topography of the surface of samples made using SLM technology, where the material is sintered layer by layer, the impact of technological parameters may be more visible just for the surface waviness. Moreover, in many scientific studies wave is ignored. Hence the novelty and purposefulness of the research presented in this article.

Such analysis can be carried out with the use of a number of methods [26], among others, analysis of surface texture parameters or multiscale analysis [27,28,29,30].

In the paper [31], the authors carried out research on models made with the use of selective laser melting technology and AlSi10Mg powder. The main technological parameter, the impact of which was analyzed, was the angle of inclination of the model plane in relation to the construction platform (the so-called printing orientation). The measurement of dimensional accuracy of selected geometrical features of the model was also carried out.

In [32], the authors conducted an analysis of the dimensional and shape accuracy of models produced using DMLS technology using the EOSINT M270 machine and material GP1 based on metal powder. In addition, an analysis of the quality of the geometric structure of the surface was carried out by determining selected roughness parameters. The work also used finishing machining through the use of CNC machining. In addition, the tolerance class of the models thus manufactured was determined.

The research consisting in the analysis of the quality of the geometric structure of the surface (mainly roughness parameters) of the models made with the use of metal powders in SLM technology have been described in several research papers, including [33]. In the presented paper, the authors analyzed the geometric structure of the surface of the samples made with variable directions of layer production in relation to the construction platform. The research included materials such as: nickel alloy, aluminum alloys, stainless steels, and titanium alloys, widely used in technologies such as SLM and SLS.

The dynamic development of additive technologies has resulted in the development of new materials, especially on the basis of metal powders. In the case of technology used in work-SLM, 316L steel with high corrosion resistance is very widely used. By combining 3D printing technology (SLM) and 316L steel, it is possible to build models whose use is very large. Particularly interesting seems to be the use of such a combination of technology and materials in the aviation industry (impeller, blade, cooling channel), which is widely associated with the concept of “LEAN manufacturing”. In addition, SLM technology is very widely used in the medical industry as an alternative to constructing surgical instruments by conventional methods.

Knowledge of the impact of technological parameters, in this case the printing orientation, may allow the production of models for the medical and aviation industries with very complex geometry, impossible to produce with other conventional technologies and with high corrosion resistance. Production of models using 3D printing (SLM) allows for the manufacturing of very geometrically complex surfaces from known freeform surfaces. By knowing about the 3D printing process, we are able to predict the surface quality (roughness and waviness) of models and their wear process, because the waviness in the wear process plays a key role.

On the basis of a literature review and preliminary research results it can be stated that additive technologies are and will be an excellent alternative to conventional manufacturing technologies, however, there are no standards and studies describing the influence of technological parameters on the quality of the geometric structure of the surface. Therefore, it can be considered reasonable to discuss in the presented paper the topic of determining the impact of the orientation of model positioning on the construction platform on the waviness parameters of the geometric structure of the surface.

## 2. Materials and Methods

**Figure 1:**
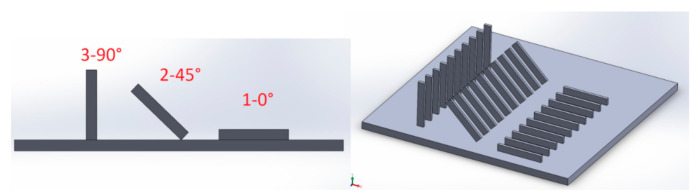
Arrangements (orientations & positioning) of investigated samples on the experimental platform.

The test samples were designed using SolidWorks software. Then the designed 3D CAD models were saved as .stl files with the following approximation parameters: linear tolerance of 0.005 and angular tolerance of 5°. Next, the models were placed on a virtual construction platform with three preset values of the inclination angle with respect to the construction platform. The view of the samples on the virtual platform is shown in Figure 1. For each print variant, the measurement planes of the samples were in the same plane of the coordinate system, i.e., in the X-Z axes. The following figure shows the CAD model and the saved .stl format with the measurement plane marked, where the measurement of waviness parameters was carried out. For the construction of the sample models, a well-known corrosion-resistant steel-based structural material (316L) was used. Samples were made in the number of 30 pieces (10 for each printing orientation).

**Figure 2:**
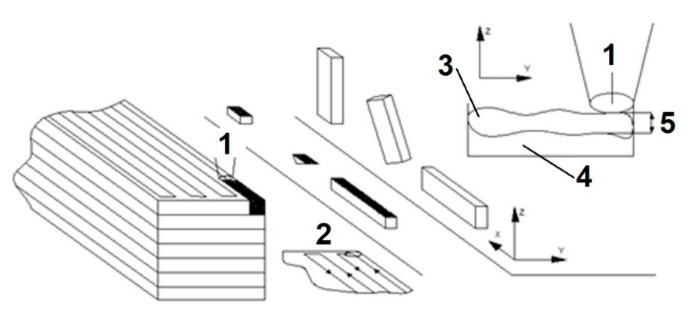
Schematic principle of SLM technology (scales are not respected), where: (**1**) laser beam; (**2**) laser path; (**3**) melted power; (**4**) powdered bed; (**5**) layer thickness.

3D printing, SLM technology is based on additive layers of material, and then local remelting with a laser, which is a source of energy. In this technology, the powder layer after spreading on the platform with a laser is heated locally, which melts the material and connects to the previously formed layer. The whole process takes place in an inert atmosphere-nitrogen gas. The principle of SLM technology was shown in Figure 2.

The CONCEPT LASER M2 machine (3D printer) was used to construct the sample models. The machine has a working chamber with the dimensions of 250 × 250 × 350 mm^3^ and is equipped with a laser system with a power of 2 × 200 W. The machine allows you to build models using many materials, such as: 316L, CL 30/31AL, titanium, bronze and nickel-based powders, especially for dental applications. After completion of the construction process, the samples were subjected to heat treatment by annealing. The furnace was heated to 550 °C for 3 h, and then heated for 6 h, and then cooling was conducted with the furnace. The thickness of the constructed layer was 25 μm. The overage grain size was equal in range 19–30 µm. Printing parameters were: laser power-200 W, scan speed 1600 mm/s and protective chamber atmosphere nitrogen gas. Apparent density of 316L powered is 4–4.5 g/cm^3^ at 20 °C. Moreover, for this technology density of sintered models is around 99.6%.

**Figure 3:**
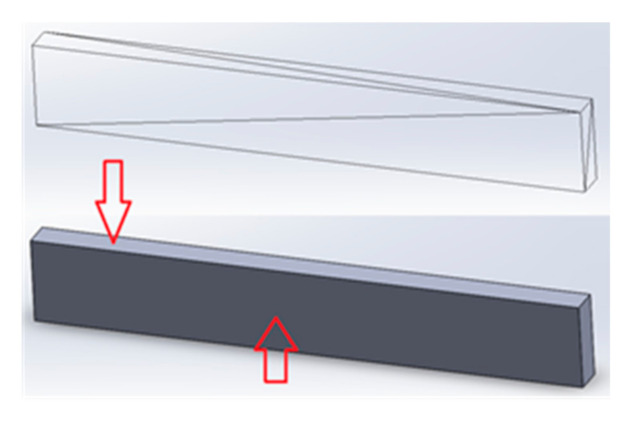
Examined surfaces.

Measurements of the geometric structure of the surface can be carried out using a number of methods. The tests and determination of the parameters of the geometric structure of the surface were carried out in accordance with the applicable standards [34,35]. The optical profiler Talysurf CCI Lite with Mirau interferometer was used to measure the lateral and frontal surfaces (Figure 3) using a 10× zoom lens. A matrix of measurement points in the size 1024 × 1024, which corresponds to a field with the dimensions of 1.66 mm × 1.66 mm, was obtained. In order to determine the surface waviness parameters, the obtained surfaces were leveled, the examined matrix was transformed into a series of profiles (a series of 1024 profiles was obtained for testing), and then the filtration process was carried out with the use of a Gauss filter (cut-off 0.8 mm). In case of using advanced optical measuring instruments, an appropriate (adequate) model and “a priori information” it is able to obtain results with higher bandwidth [36] The authors analyzed 2D profiles because in case of full-knowledge of the surface texture and its characteristics the examination of a single profile is sufficient to draw utilitarian conclusions and for control process. The mechanical properties and chemical composition of steel declared by the machine manufacturer are presented in Table 1 and Table 2.

The following parameters characterizing the waviness of the surface were analyzed:

*Wa*-the arithmetic mean of the waviness profile deviations, which is defined as the arithmetic mean of the absolute ordinate values calculated from the dependencies:

(1) $$Wa=\frac{1}{N}\sum_{i=1}^{N}\left|z_{i}\right|$$

where: *N*-number of measurement points of the profile;

*Wt*-total height of the waviness profile, which is defined as the sum of the height of the highest elevation of the *Zp* profile and the highest depth of the *Zv* elevation calculated from the formula:

(2) $$Zp=\sup(z_{i})\;Zv=|\inf(z_{i})|\;Wt=Zp+Zv$$

*Wsk*-the slope of the waviness profile, which is defined as the quotient of the mean value of the third power of the third power of the *z_i_* ordinates and the third power of the *Wq* respectively, along the length of the elementary section, calculated from the formula:

(3) $$Wsk=\frac{1}{Wq^{3}}\frac{1}{N}\sum_{i=1}^{N}z_{i}{}^{3}$$

where: *Wq*-the square mean of the waviness deviations, which is defined as the square mean of the ordinates of the *z_i_* profile calculated from the formula:

(4) $$Wq=\sqrt{\frac{1}{N}\sum_{i=1}^{N}z_{i}{}^{2}}$$

*Wku*-a kurtosis of the waviness profile, which is defined as the quotient of the average of the fourth order of the *z_i_* ordinate values and the *Wq* fourth power calculated from the formula:

(5) $$Wku=\frac{1}{Wq^{4}}\frac{1}{N}\sum_{i=1}^{N}z_{i}{}^{4}$$

The parameters selected to measure the surface waviness are amplitude parameters. The inclination of the waviness profile *Wsk* is a measure of the asymmetry of the probability density function of the ordinate values, while the kurtosis of the waviness profile is a measure of the slope of the probability density function of the ordinate values.

## 3. Results and Discussion

The calculation of the individual waviness parameters described by the Equations (1)–(5) is performed by the computer system of the optical profiler Talysurf CCI Lite according to the appropriate algorithms. To provide complex information about the waviness of the surface of elements made using the SLM technology (powder bed fusion), the waviness of each sample’s frontal and lateral surface was examined. The results of measurements of waviness parameters (Wa, Wt, Wsk and Wku) for the front surfaces of the analyzed samples are presented in Table 3.

Samples numbered 1.1 to 1.10 were made at an angle of 0° to the construction platform, samples 2.1 to 2.10 at 45° and samples 3.1 to 3.10 at 90°. In order to facilitate the interpretation of the measurement results, the following were calculated: mean values ($\bar{x}$), square mean errors (s) and ranges (R = x_max_ − x_min_).

The preliminary analysis of the test results presented in Table 3 has shown that the average values of the waviness parameters for all analyzed samples remain at a similar level (regardless of the printing orientation chosen). However, when considering amplitude parameters such as Wa and Wt, we can see that the lowest values of these parameters were obtained for samples mad at an angle of 0° and the highest for samples made at an angle of 45°. However, the average values of amplitude parameters for samples placed on the construction platform at 45° and 90° angles are insignificant. When analyzing the values of the mean square error and range, which were calculated for the Wa and Wt parameters for individual samples made at an angle of 90° (samples no. 3.1–3.10), it can be concluded that there are significant discrepancies between the tested waviness parameters. This may result from the layered structure of the tested elements. In the case of the samples numbers 3.1 to 3.10, the subsequent layers of material were laid in a direction perpendicular to the measured surface, which may affect the divergence of the measurement results. It should be added that the value of the deviation of the ordinates of the waviness profile Wa is important for lubrication and sealing. Therefore, the 0° print orientation is recommended for the production of such elements.

**Figure 4:**
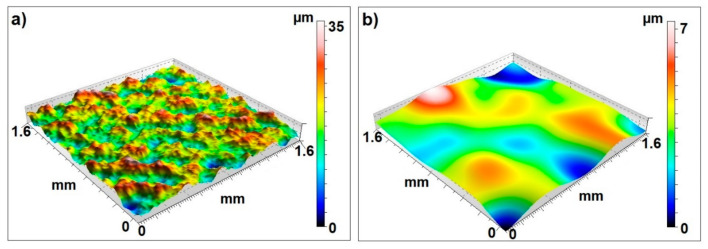
Frontal 3D surface topography: (**a**) surface before filtering, (**b**) surface waviness.

Analyzing the parameters describing in more detail the character of the surface irregularities, namely the kurtosis of the Wku profile and the inclination of the Wsk waviness profile, it can be stated that many small and more rounded peaks and grooves occur on the waviness profile. For the waviness parameter Wku less than 3 the distribution of surface irregularities trends to regular distribution, while for Wsk value is higher than 3 the sample surface is smooth without many peaks and valleys. Moreover, the value of the Wsk parameter that is close to zero means that the distribution of waviness of the tested samples is similar to the normal distribution, which is characteristic for symmetrical profiles [37,38]. These values are lower than zero for surface characterized by smooth surfaces with deep scratches. For Wsk values higher than zero there is a lot of deep valleys and small number of peak. Figure 4a shows an example of the topography of the frontal surface of a tested sample before filtration and Figure 4b shows the 3D waviness.

**Figure 5:**
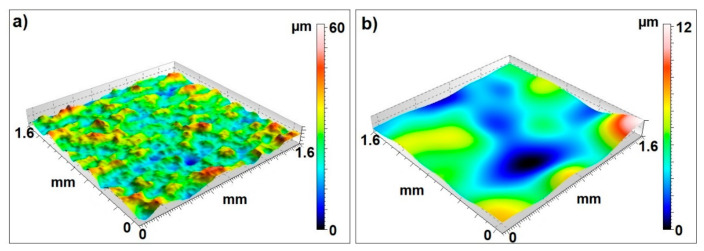
3D Lateral surface topography: (**a**) surface before filtering, (**b**) surface waviness.

Analyzing the test results presented in Table 4, it can be unequivocally stated that the values of Wa and Wt waviness parameters measured for the lateral surface exceed the waviness parameters measured at the frontal surface. However, for the lateral surface, the highest values of waviness parameters were obtained for samples printed at an angle of 0°. It should be added that for the same print orientation, the values of Wa and Wt parameters were the smallest on the front surface (see Table 3). The lowest values of Wa and Wt waviness parameters were obtained for samples manufactured at an angle of 90°. In addition, the waviness parameters measured for the lateral surface are more divergent, as confirmed by the calculated mean square error values (see Table 4). Analyzing the parameters describing the skewness of the waviness profile (Wsk) and the kurtosis (Wku), similar conclusions can be drawn as in the analysis of the frontal surface waviness. There are also many small and rounded peaks in this case. In addition, the distribution of the waviness of the lateral surface is close to normal. Similarly to the front surface, the topography of the lateral surface before filtration is shown in Figure 5a, and the waviness of the lateral surface is shown in Figure 5b.

When analyzing the overall results of waviness measurements, it can be stated that it is important to consider the function of the given surface before selecting the SLM print orientation. When creating elements where the frontal and lateral surfaces will play important functions and obtaining low waviness is recommended, it would be a good compromise to print the elements at an angle of 45°. For such placement of samples on the 3D printer construction platform, it allows for relatively fast printing and satisfactory waviness of both frontal and lateral surfaces.

In addition, for all 30 samples, both for the frontal and lateral surfaces, overall statistical parameters such as the mean value $\bar{x_{c}}$ and the SDc standard deviation were calculated. The standard deviation was calculated using the formula:

(6) $$SD_{c}=\sqrt{\frac{1}{\left(n-1\right)}\sum_{i=1}^{n}{\left(x_{i}-{\bar{x}}_{c}\right)}^{2}}$$

where: *n*-sample size, $\bar{x_{c}}$–arithmetic mean of all the measured values in the sample.

With the expected value and the standard deviation, we can present the results in the form of a normal distribution based on the formula:

(7) $$f(x)=\frac{1}{SD_{c}\sqrt{2\pi }}e^{\frac{-{\left(x-{\bar{x}}_{c}\right)}^{2}}{2SD_{c}{}^{2}}}$$

where: *f(x)*-probability density function.

**Figure 6:**
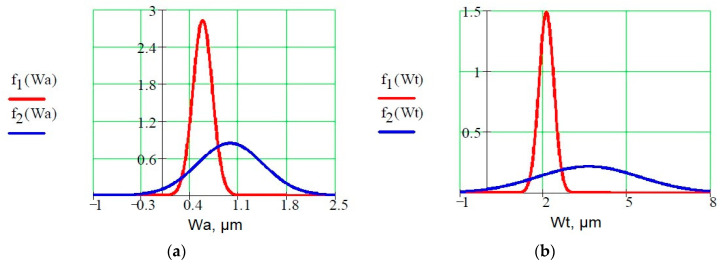

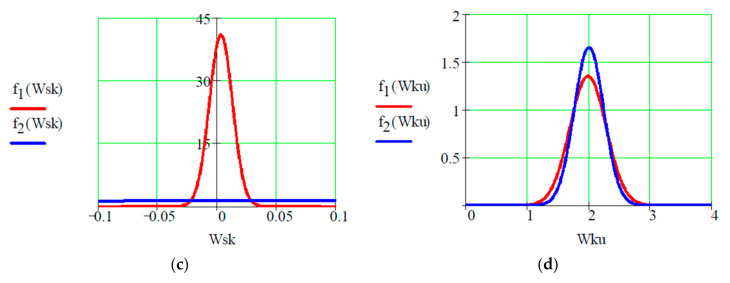
Normal distribution of probability densities; f1(Wa), f1(Wt), f1(Wsk), f1(Wku)-probability densities of the individual waviness parameters measured on the frontal surfaces of the samples (red line), f1(Wa), f1(Wt), f1(Wsk), f1(Wku)-probability densities of the individual waviness parameters measured on the lateral surfaces of the samples (blue line); (**a**) normal distributions for the waviness parameter—Wa, (**b**) normal distributions for the waviness parameter-Wt, (**c**) normal distributions for the waviness parameter—Wsk, (**d**) normal distributions for the waviness parameter-Wku.

Figure 6 presents an example of normal distributions of probability density functions for each of the waviness parameters measured on the frontal surfaces (red line) and lateral surfaces (blue line) of the samples.

Analyzing the probability density distributions for individual waviness parameters, it can be concluded that the value of Wsk inclination for frontal surfaces is close to zero and there is no large dispersion. The other parameters do not show a large dispersion in the frontal surface of the samples. This is a positive sign for this manufacturing technology. The waviness created as a result of additive shaping is regular.

In order to better assess the influence of the printing orientation on the waviness of the surface, relative values of individual parameters expressed as a percentage have been introduced. The mean value of a given parameter obtained on the basis of waviness measurements of samples for all orientation of printing was used as the basis—in this case it is the mean of thirty samples. The mean values of the individual waviness parameters and the standard deviation for the frontal and lateral surfaces of 30 samples are shown in Table 5.

The mean value of the parameter obtained from measurements for each printing orientation was related to the mean value, obtaining relative values of surface parameters for individual printing orientations. This is described by the formulas:

(8) $$\Delta W_{0}=\frac{\bar{|W}-{\bar{W}}_{0}|}{\bar{W}}\times 100\%$$

(9) $$\Delta W_{45}=\frac{\left|\bar{W}-{\bar{W}}_{45}\right|}{\bar{W}}\times 100\%$$

(10) $$\Delta W_{90}=\frac{\bar{|W}-{\bar{W}}_{90}|}{\bar{W}}\times 100\%$$

where: $\Delta W_{0}$, $\Delta W_{45}$, $\Delta W_{90}$-relative values of surface parameters for individual print orientation, $\bar{W}$-mean value of the waviness parameter for all orientation of printing (thirty samples), $\Delta {\bar{W}}_{0}$-mean value of measured waviness parameters for the print orientation 0°, $\Delta {\bar{W}}_{45}$-mean value of measured waviness parameters for the 45° print orientation, $\Delta {\bar{W}}_{90}$-mean value of measured waviness parameters for the 90° print orientation.

For example, the relative value of the arithmetic mean of the waviness profile deviations of the frontal area for the print orientation 0° calculated from the Equation (8) on the basis of the data from Table 3 and Table 4 amounts to:

(11) $$\Delta Wa_{0}=\frac{\bar{Wa}-{\bar{Wa}}_{0}}{\bar{Wa}}\times 100\%$$

where: $\Delta Wa_{0}$-relative arithmetic mean of the waviness profile deviations for the print orientation 0°, $\bar{Wa}$-arithmetic mean of the waviness profile deviations for all orientation of printing (on the basis of Table 5 it is 0.58), ${\bar{Wa}}_{0}$-arithmetic mean of the waviness profile deviations for the print orientation 0° (on the basis of Table 3 it is 0.44).

**Figure 7:**
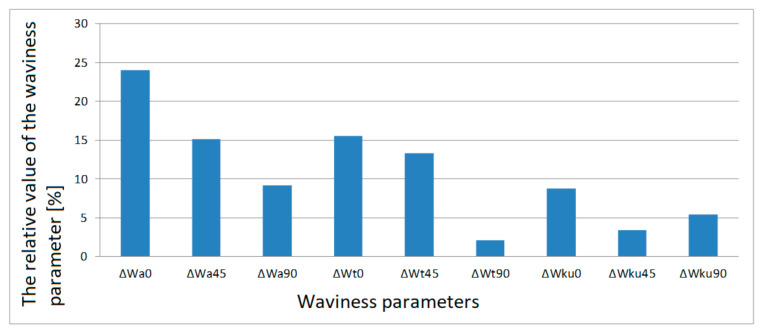
Relative values of frontal surface waviness parameters for each print orientation.

**Figure 8:**
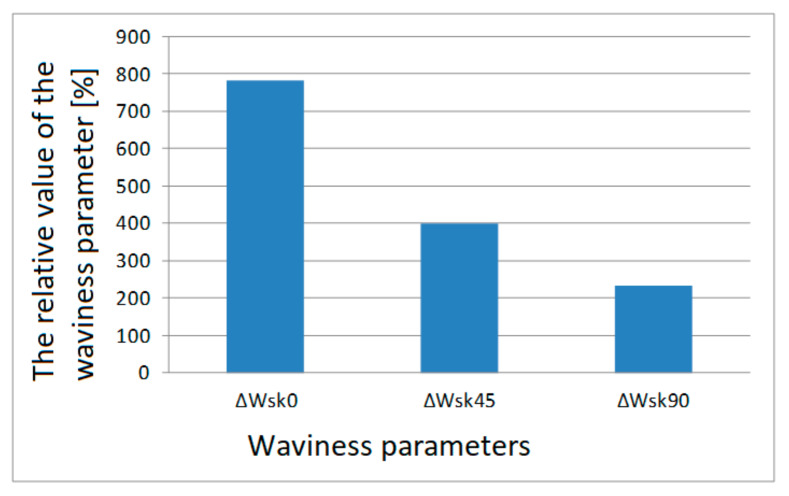
Relative values of the waviness parameter Wsk of the frontal surface for each print orientation.

**Figure 9:**
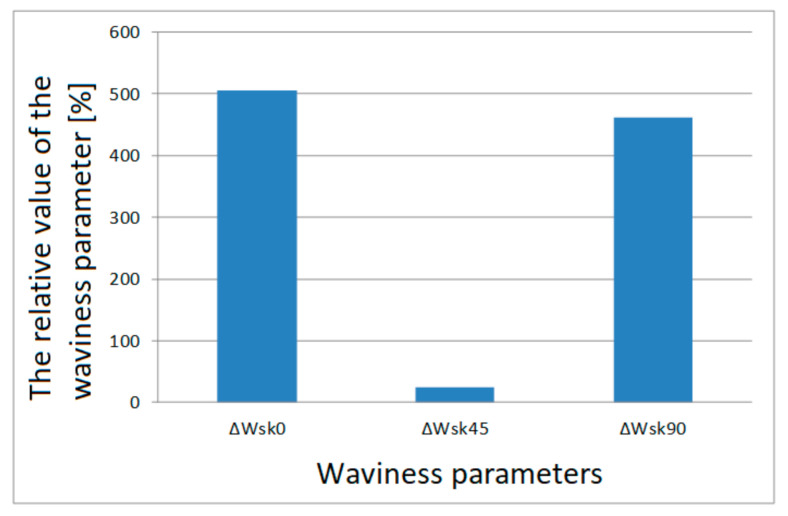
Relative values of lateral surface waviness parameters for each print orientation.

**Figure 10:**
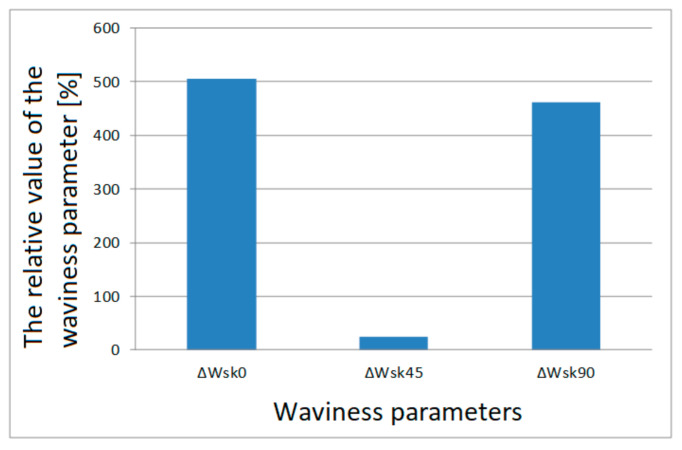
Relative values of the waviness parameter Wsk of the lateral surface for each print orientation.

The value of $\Delta {\bar{Wa}}_{0}$ calculated using Formula (11) is 24.14%. In this way, all the relative values of the surface parameters in relation to the printing orientations were calculated. Relative values of individual parameters are presented in column charts, Figure 7, Figure 8, Figure 9 and Figure 10.

Relative values of the Wsk waviness parameter for individual printing orientations are shown on a separate graph due to large percentage differences for this parameter. These large percentage differences result from the specificity of the Wsk parameter, which includes the Wq waviness parameter, which is the square mean of the waviness deviations.

If we analyze Figure 7 and Figure 8, we can observe a certain regularity. For all the tested waviness parameters, the largest relative differences apply to the surface area of samples printed in the 0° orientation. In absolute terms, this is not important in this particular case, but the information is important if we want to print a specific model. The smallest waviness is reached on surfaces arranged in the 0° orientation.

For the lateral surfaces, the distribution of the relative waviness parameters is different than for the frontal surfaces, as shown in the diagrams in Figure 9 and Figure 10. The smallest relative value of waviness parameters was found for samples printed at an angle of 45°.

## 4. Conclusions

The following conclusions can be drawn from the analysis of the presented results of tests of the surface of samples made with powder bed fusion technology and 316L steel:

1. The waviness of the surface of models manufactured using laser additive technology of sintering of metal powders in the case of selected groups of parameters is clearly dependent on the orientation of printing (orientation of parts on the working platform). Analyzing the values of the frontal surface waviness parameters (Wa, Wt), almost in all cases the values of the parameters increased with the increase of the angle of inclination of the examined plane in relation to the construction platform.
2. The additive technology of laser sintering of metal powders results in surfaces with quite regular waviness and small waviness deviations. Therefore, it can be said that this technology can be used to build fully functional models with complex geometry. Moreover, regular waviness and small deviations obviously have a positive effect on the functional properties of the models produced, such as tribological. The results of the research can be used in science and in design stage and implementation works through the possibility of adapting the research results (parameters of the surface layer) in order to conduct computer simulations at the implementation and design stage. The technological parameters of the printing direction in almost all laser technologies using metal powders will affect the waviness parameters in an analogous way, which allows the results of the research to be transfered into other 3D printing technologies and powder materials.
3. Spatial analysis of surface texture showed that surfaces are characterized by a significant number of rounded peaks. The surface with similar characteristics of unevenness shows lower friction coefficient values, which confirms the value of Wku and Wsk parameters.
4. All measured waviness parameters on the lateral surfaces of the tested samples differ substantially from those measured on the frontal surfaces, which is clearly shown in quantitative terms on the curves in Figure 6a–c and in qualitative terms in Figure 4 showing the surface topography. These differences result from the technological process itself, or more precisely from the position of the surface of rectangular samples in relation to the direction of the laser beam melting the powder. The frontal surfaces of the samples were always aligned parallel to the direction of the laser beam. No significant differences were found in the Wku parameter (Figure 6d) kurtosis of the waviness profile, which results from the very definition of this parameter, because in the formula for its calculation there appear fourth powers of *z_i_* ordinates and fourth powers of Wq, and this eliminates differences in the obtained curves of probability density functions. This characteristic can be directly attributed to the different ways in which the sample planes (frontal and lateral) are created. In case of two frontal surfaces (0°, 45°) the hatching phenomenon occurred, but in the case of all lateral surfaces and one frontal surface (90°) there was no such phenomenon.
5. It seems reasonable to use several parameters to assess surface waviness, because only then can a more complete evaluation of the quality of the elements produced using additive technology be obtained.

## Figures and Tables

**Table 1:** Chemical composition of 316L material.

Component	Cr	Ni	Mo	Mn	Si	P	C	S	Fe
Indicative value, %	16.5–18.5	10.0–13.0	2–2.5	0–2.0	0–1.0	0–0.045	0–0.030	0–0.030	Balance

**Table 2:** Mechanical properties of 316L steel.

Properties	90°, Upright	45°, Polar Angle	0°, Horizontal
Yield strength R_p0,2_^1^	374 ± 5 N/mm^2^	385 ± 6 N/mm^2^	330 ± 8 N/mm^2^
Tensile strength Rm ^1^	650 ± 5 N/mm^2^	640 ± 7 N/mm^2^	529 ± 8 N/mm^2^
Elongation A ^1,2^	65 ± 4%	63 ± 5%	63 ± 5%
Young’s modulus ^3^	ca. 200 × 10^3^ N/mm^2^	ca. 200 × 10^3^ N/mm^2^	ca. 200 × 10^3^ N/mm^2^
Thermal conductivity λ ^3^	ca. 15 W/mK	ca. 15 W/mK	ca. 15 W/mK
Hardness ^4^	20 HRC	20 HRC	20 HRC

^1^ Tensile test at 20 °C according to DIN EN 125; ^2^ Using appropriate heat treatment a higher elongation can be achieved; ^3^ Specification according to the material manufacturer’s data sheet; ^4^ Hardness test according DIN EN ISO 6508.

**Table 3:** Waviness parameters versus orientations for the frontal surface.

Orientation & Ref		Wa, µm	Wt, µm	Wsk	Wku	Orientation & ref.		Wa, µm	Wt, µm	Wsk	Wku	Orientation & Ref.		Wa, µm	Wt, µm	Wsk	Wku
1 (0°)	1.1	0.53	2.18	0.07	2.18	2 (45°)	2.1	0.52	1.95	−0.11	2.13	3 (90°)	3.1	0.51	1.69	−0.11	1.53
	1.2	0.51	1.92	0.21	1.85		2.2	0.68	2.75	−0.48	2.47		3.2	0.74	2.43	−0.15	1.78
	1.3	0.47	1.83	0.22	2.38		2.3	0.87	2.83	0.14	1.91		3.3	0.63	2.08	0.10	1.81
	1.4	0.28	1.57	0.22	2.21		2.4	0.60	1.79	−0.02	1.60		3.4	0.29	1.36	0.22	2.32
	1.5	0.27	1.10	0.06	2.07		2.5	0.82	2.91	0.08	1.87		3.5	0.61	2.37	0.20	2.60
	1.6	0.21	0.89	−0.09	2.24		2.6	0.59	2.14	0.29	1.79		3.6	0.41	1.47	−0.17	1.80
	1.7	0.28	1.32	−0.03	2.26		2.7	0.82	3.04	0.07	1.90		3.7	0.99	3.30	−0.01	1.60
	1.8	0.71	2.69	−0.30	2.02		2.8	0.58	2.10	−0.04	1.69		3.8	0.92	2.77	0.21	1.62
	1.9	0.76	2.93	−0.26	2.52		2.9	0.52	1.70	0.04	1.67		3.9	0.60	1.88	−0.40	1.79
	1.10	0.34	1.30	0.18	1.84		2.10	0.62	2.59	−0.06	2.13		3.10	0.58	2.10	0.20	1.91
$\bar{x}$		0.44	1.77	0.03	2.16	$\bar{x}$		0.66	2.38	−0.01	1.92	$\bar{x}$		0.63	2.15	0.01	1.88
s		0.19	0.67	0.20	0.22	s		0.13	0.50	0.20	0.26	s		0.21	0.60	0.21	0.33
R		0.55	2.04	0.53	0.68	R		0.35	1.34	0.77	0.87	R		0.70	1.94	0.62	1.07

**Table 4:** Waviness parameters versus orientations for the lateral surfaces.

Orientation & Ref.		Wa, µm	Wt, µm	Wsk	Wku	Orientation & ref.		Wa, µm	Wt, µm	Wsk	Wku	Orientation & ref.		Wa, µm	Wt, µm	Wsk	Wku
1 (0°)	1.1	1.56	5.02	0.06	1.98	2 (45°)	2.1	0.72	2.32	0.30	1.73	3 (90°)	3.1	0.64	2.05	−0.04	1.72
	1.2	1.14	4.09	−0.02	1.93		2.2	0.71	2.84	−0.53	2.18		3.2	0.74	2.28	0.20	1.60
	1.3	0.98	3.75	−0.23	2.12		2.3	2.29	9.46	0.28	2.07		3.3	0.67	2.51	0.44	2.14
	1.4	1.92	5.84	0.11	1.49		2.4	0.62	2.23	0.30	2.23		3.4	0.62	2.41	−0.20	1.86
	1.5	1.02	4.09	−0.34	2.29		2.5	0.76	2.97	0.30	2.13		3.5	0.52	2.47	0.28	2.34
	1.6	1.30	5.57	0.38	2.29		2.6	0.54	2.25	−0.06	2.24		3.6	1.05	3.45	0.35	1.84
	1.7	1.47	6.63	−0.31	2.16		2.7	0.48	1.69	−0.21	1.91		3.7	0.89	2.68	0.47	1.69
	1.8	1.81	6.45	−0.13	2.03		2.8	0.43	1.57	0.04	1.76		3.8	0.74	3.03	−0.36	2.42
	1.9	1.46	5.62	−0.23	2.06		2.9	0.56	2.20	0.11	1.84		3.9	0.45	1.56	0.20	1.74
	1.10	1.04	4.10	−0.55	2.32		2.10	1.23	4.48	-0.08	1.93		3.10	0.87	2.96	0.43	1.89
$\bar{x}$		1.37	5.12	−0.13	2.07	$\bar{x}$		0.83	3.20	0.04	2.00	$\bar{x}$		0.72	2.54	0.18	1.92
s		0.33	1.06	0.27	0.24	s		0.56	2.35	0.27	0.19	S		0.18	0.53	0.29	0.28
R		0.94	2.88	0.93	0.83	R		1.86	7.89	0.82	0.51	R		0.60	1.89	0.83	0.82

**Table 5:** Mean values of surface waviness parameters for 30 measurements for frontal and lateral surfaces.

Waviness Parameters versus Orientation		Wa, µm	Wt, µm	Wsk	Wku
Frontal surface	$\bar{W}$	0.58	2.1	0.003	1.98
	SD	0.14	0.27	0.01	0.30
Lateral surface	$\bar{W}$	0.97	3.62	0.03	2
	SD	0.47	1.84	0.30	0.24

## Nomenclature and Abbreviations

SLS	Selective Laser Sintering
FDM	Fused Deposition Modeling
FFF	Fused Filament Fabrication
LOM	Laminated Object Manufacturing
3DP	3D printing
DMLS	Direct Metal Laser Sintering
SLM	Selective Laser Melting
FEF	Freeze-Form Extrusion Fabrication
R_p0,2_^1^	Yield strength
Rm	Tensile strength
A	Elongation, %
Λ	Thermal conductivity
Wa	the arithmetic mean of the waviness profile deviations, µm
Wt	total height of the waviness profile, µm
Wsk	the slope of the waviness profile
Wq	the square mean of the waviness deviations, µm
Wku	a kurtosis of the waviness profile
$\bar{x}$	mean value
s	square mean errors
r	range
*SDc*	standard deviation
*n*	sample size
$\bar{x_{c}}$	arithmetic mean of all the measured values in the sample
$\bar{W}$	mean value of the waviness parameter for all orientation of printing (thirty samples)
$\Delta {\bar{W}}_{0}$	mean value of measured waviness parameters for the print orientation 0°
$\Delta {\bar{W}}_{45}$	mean value of measured waviness parameters for the 45° print orientation
$\Delta {\bar{W}}_{90}$	mean value of measured waviness parameters for the 90° print orientation
